# The Editor's Letter-Box

**Published:** 1919-01-04

**Authors:** 


*To the Editor of* THE HOSPITAL.

SIR,—In the issue of December 21 "Paul Ray" says "surely there are some hospitals where they have found a way for keeping day duties to the day." Here is an instance: In Anson Hospital (c.z. Isthmus of Panama) we did not start in the "middle of the night." Night nurses only nursed those who were so ill as to need attention, the others were not disturbed between 9 P.M. and 6 A.M.

Temperatures were taken, beds made, etc., when the day people came on. Day orderlies came at 6 A.M. and day nurses at 7 A.M. Orderlies were on duty twelve hours, with half-day once a week and three hours on Sunday off. Day nurses eight hours duty, night nurses ten hours; no regular off-duty days. The arrangement was certainly admirable for patients and the work. Sometimes we did wish all eight-hour duties were consecutive hours.

However, this was the arrangement. A section—anywhere from 200 to 300 beds, usually two acute and two convalescent wards—had eight nurses assigned to it. One section nurse, hours 7 to 12—3 to 6, two broken hours, nurses same hours as section nurse, two three o'clock hours, 7 to 3, and two nine o'clock hours, 7 to 9 and 3 to 9; one night nurse, 9 P.M. to 7 A.M. Orderlies were about in the proportion of two for each nurse, never less. All the nurses were fully qualified, and the duties changed once a month, with the exception of section nurse, which was often held for four to six months, owing to the fact that she looked after stores, linen, etc. Only patients on the danger list and those calling an orderly were disturbed between ten and six, and night nurses were required to walk through every ward at least once an hour. Patients very ill were kept in small wards and side room as much as possible. I am quite safe in saying that nine nights out of ten any patient able to sleep had at least seven hours without being disturbed, often more.

"ANCON."

